# Study on the occurrence law and green control of grape gray mold from the perspective of ecological balance

**DOI:** 10.1080/21655979.2021.1888578

**Published:** 2021-02-28

**Authors:** Fengying Shen, Weigang Wu, Xing Han, Jiao Wang, Yaning Li, Daqun Liu

**Affiliations:** a College of Plant Protection, Agricultural University of Hebei/Innovation Center for Biological Control of Crop Diseases and Insect Pests of Hebei Province/National Engineering Research Center for Agriculture in Northern Mountainous Areas, Baoding 071001, China; b College of Agriculture and Forestry Science and Technology of Hebei North University, /Hebei Key Laboratory of Quality & Safety Analysis-Testing for Agro-Products and Food, Hebei North University, hangjiakou 075000, China; c Graduate School, Chinese Academy of Agricultural Sciences, Beijing, China

**Keywords:** Ecological balance, *Botrytis cinerea*, incidence regularity, prevention and cure

## Abstract

With the increase of grape planting years, the base number of pathogenic seedlings and insect population is gradually rising. In addition, the introduction, breeding system and control of seedlings are not standardized and other human factors, the occurrence of *Botrytis cinerea*(*B.cinerea*) on grape is becoming more and more serious, resulting in a prominent problem of yield decline. In this paper, the occurrence of *B.cinerea* was monitored and its control effect was tested from the perspective of ecological balance. Finally, the biological characteristics and control of *B.cinerea* were studied. The spore catcher was used to catch the pathogen spores of *B. cinerea*, and the amount of sporangium scattering reached its peak from August to September Spore scattering is affected by meteorological factors, and the temperature has reached a very significant level, and the low temperature and high humidity conditions are conducive to the disease; The results showed that the resistance frequency of 304 *B*.*cinerea*strains to *carbendazim, boscalid, pyrimethanil* was higher than 50%; the volatile compounds produced by yeast (*Trichosproom* sp.) YE-3-2 significantly inhibited the growth of *B.cinerea* (inhibition rate was 62.93%, according to the occurrence regularity of *B.cinerea*, the accurate and effective agricultural measures had a good control effect on *B.cinerea*, which could improve the quality of grape fruit and provide some help for the prevention of grape gray mold.

## GRAPHICAL ABSTRACT

**Figure:**
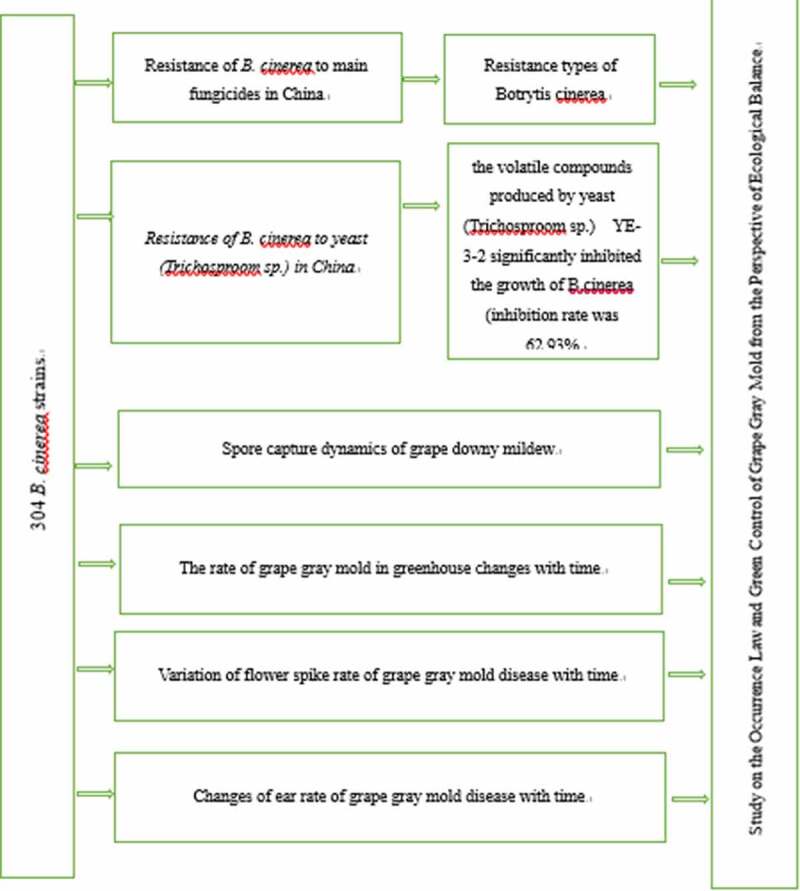

## 1. Introduction

The prevention and control of gray mold will not only cause pollution of fruits and environment, but also make pathogen population prone to drug resistance. *B.cinerea* has the characteristics of wide host range, rapid genetic variation and rapid spread, which makes it occurs in different periods such as cultivation period, post-harvest transportation, and storage, and brings serious losses to grape industry [1]. Because it is an old grape producing area, it is difficult to prevent and cure the disease, and the recurrence rate is high. In severe cases, it will cause no harvest. Grapes are in a period of high humidity and fog from germination stage to young fruit stage, which leads to a long period of grape gray mold and a large loss, and the field incidence rate is between 20% and 30%, which poses a serious threat to the development of grape industry.

*B. cinerea* is a devastating disease on vines, which causes direct loss of grape yield of about 20%-30% every year, and can reach 50% of the total yield in severe cases [2]. *B. cinerea* seriously affects the yield and quality of grapes and restricts the development of grape economy [3]. However, the long-term extensive application of agricultural drugs makes the resistance of pathogenic bacteria to drugs gradually increase, and finally, it is difficult to effectively control the occurrence of diseases. The occurrence of grape diseases will not only affect the quality and yield of grape fruit, but also pose a risk to the safety of grape and wine, seriously threaten human health and pollute the ecological environment to a certain extent. The prevention and control of wine grape diseases and insect pests has become an urgent problem to be solved.

Grape mold is a major disease in grape production, which is mainly caused by *B.cinerea*. As long as the disease occurs in a suitable environment, it will cause serious damage to young leaves, flower buds and fruits of grapes. Therefore, mastering the species and occurrence regularity of grape diseases is of great significance to effectively control grape diseases , improve grape quality and yield, and stabilize wine industry. In this study, disease-resistant grapes were used as test materials from the perspective of ecological balance.

The purpose of this paper is to explore the ecological control, biological control, physical control, and other environmental friendly measures to control the behavior of pests by detecting the resistance of 304 strains of *B.cinerea* to commonly used fungicides, the inhibition rate of yeast (*trichosproom* sp.) YE-3-2 to *B. cinerea*, and the occurrence regularity of *B. cinerea* in greenhouse. Starting from the whole farmland ecosystem, based on agricultural control, we should actively protect and utilize natural enemies, improve the disease resistance and insect resistance of crops, and use them reasonably when necessary Chemical pesticides can reduce the damage of diseases and insect pests to the minimum.

## 2. Symptomatic characteristic

Grape gray mold blight is the serious disease threatening grape production during flowering phase, young fruit stage, veraison, maturation stage, and storage period. After the inflorescence became infected, the damaged part produced light brown and watery spots at the initial stage, and then turned dark brown and soft rot. When the weather is wet, a layer of rat gray mold is produced in the affected area, which is the conidiophore and conidia of pathogenic bacteria. The young branches were damaged, and light brown spots of irregular shape appeared. When the disease spots were not prevented in time, rat gray mold layers would be produced, and the later disease sites were bleached white, covered with black sclerotia or gray conidia hyphae. a layer of rat gray mold is produced in the affected area, which is the conidiophore and conidia of pathogenic bacteria. The disease on the leaves starts from the tip of the leaf. It spreads inward in a “V„ shape along the veins, gray-brown, with dark and light lines on the sides, and the boundary of the diseased key is distinct the aggravation of the disease, the whole bud became sick and became brown-red necrotic, and the formed dead bud dried up on sunny days, and fell off when touched lightly.

Grapes can be infected with gray mold after sprouting and spreading leaves in spring, and the diseased buds and shoots turn brown first and finally dry up; When infected before flowering, light brown spots appeared on the edge of vein of new leaves. *B. cinerea* conidiophore is slender, branched, colorless, brown, or light gray, and the apical cells are expanded into spheres, on which many petioles are attached; The conidia are single cells, which are planted on small stems and gather into grape spikes, which are colored or gray. Mature fruit was killed, and the peel was damaged and the flesh was intact when it was lightly touched with fingers at the beginning. Sometimes, brown spots with inconspicuous rings appear, and gray mold layers are often produced at the spots. In the middle stage, sunken spots appeared on the fruit surface or cracks appeared in the pericarp of the diseased area, which quickly rotted and expanded the whole fruit, with rat gray mold layer growing on it, the stem turning brown, and soon black block sclerotia growing. After the extension, the whole ear was covered with mold layer and the fruit was completely rotted.

At the initial stage of infection, young fruits produce brown sunken spots. When new shoots and leaves are killed, light brown and irregular spots are produced. Sometimes, the disease spots appear inconspicuous rings, and can grow rat gray mold layers, and finally dry up. When the leaves are killed, light brown irregular spots are produced. Sometimes, the disease spots appear inconspicuous rings, and even when the weather is wet, rat gray mold layers can grow. Therefore, the postharvest gray mold may be directly infected at the mature stage, or it may be induced by latent infection of fruits in the early South China, which greatly increases the difficulty of controlling the disease.

## 3. Related work

Abuqamar et al. [4] studied since the 1940s, woody indicator plants have been used to detect grape virus diseases, and then studies on serum detection, herbal indicator plants detection and molecular biology detection have been carried out one after another. Agricultural control mainly includes strengthening field cultivation and management, pruning in time, ventilation and light transmission, regular removal of sick residues in vineyards, rational application of chemical fertilizers, good control of temperature and humidity in gardens, and effective control of gray mold [5]. Chemical agents commonly used in chemical control mainly include *Azoxycycline, Procymidone, carbendazim*,and other agents. If grape fruits are infected by pathogens during storage, the diseased spots will overflow with yellowish brown mucus, and the berries will gradually change color and rot, and sometimes black sclerotia will be produced on the surface of fruit stems [6].

Grapes are perennial plants, and it is very important to improve the quarantine system of seedlings and strengthen the quarantine of virus diseases of grape seedlings for the prevention and control of grape diseases and insect pests. Chen Yufei et al [7]Real-time PCR technology is used to quantitatively detect the relationship between the spore content of the pathogen of grape gray mold in the field and the disease index in the field, so as to make effective judgments for the rapid diagnosis and early warning of grape gray mold; Zhao Yongtian et al [8]. Trichoderma can be used as an alternative to chemical pesticides; Ma et al. [9] measured the resistance values of cuticle and wax layer of grape fruit by designing simple processing sensors, and the resistance values of population offspring showed continuous distribution. Han Yanli et al. [10] found that Paenibacillus polymyxa can significantly inhibit many diseases of fruits and vegetables caused by *B. cinerea. B. cinerea* is highly saprophytic and has a wide host range, and can infect each other among different host plants [11]. At present, the control of grape gray mold is mainly based on chemical control, supplemented by pollution-free control technologies such as disease-resistant varieties, agricultural control, biological control, physical control and natural products and safe compounds [5].

## 4. Methods

### 4.1. Test material

#### 4.1.1. Experimental population

304 *B.cinerea* strains, yeast (*Trichosproom sp*.) strain YE-3-2, main fungicides *carbendazim, procymidone, fludioxonil, pyrimethanil, iprodione, boscalid* and *pyrimethanil* were provided by the Laboratory of biological control, College of plant protection, Hebei Agricultural University.

The *B. cinerea* strain was inoculated on PDA medium, and then cultured at 22°C for 3 days to obtain *B. cinerea* filaments. After 10 days of culture, *B. cinerea* conidia were collected for later use. *B. cinerea* was inoculated in PDA medium, and cultured at 22°Cfor 2 d.

#### 4.1.2 *Isolation and purification of* B. cinerea

Bring the collected *B. cinerea* samples to the laboratory, dip the spores of the strains from the collected diseased fruits with sterilized inoculation needles in the ultra-clean workbench of the laboratory, and obtain single colonies on PDA plate with 1% streptomycin sulfate; Weigh 20 g potatoes, cut them into small pieces as much as possible, put them in a pot of 1 L water, and boil them for 30 min by boiling water. Filter the excess mashed potato chips with gauze, make the filtrate to 1 L, then add 20 g of glucose and 18 g of agar, mix them well, put them into a sterilization pot, and sterilize them at high temperature and high pressure for 30 min.

#### 4.1.3 Test medium

On the ultra-clean workbench, pour the prepared culture medium into a sterile Petri dish until it is cooled; Inoculate near the alcohol lamp to ensure aseptic environment, put the strain on PDA solid culture medium, and seal the Petri dish; The relative humidity was 90%, and it was cultured at 22°C in the dark for 5 days, and no *B. cinerea* spores were produced, so it was stored at 4°Cfor later use.

### 4.2. Experimental method

#### 4.2.1. *Sensitivity of* B. cinerea *to chemical fungicides and biocontrol yeast*

304 strains were screened by plate isolation and confrontation method. Firstly, the PDA culture medium with a width of about 0.5 cm in the center of the PDA plate was removed, so that the culture media on both sides were not in contact. 10 μL spores suspension with a concentration of 1 × 10 ^8^ cfu/mL was coated on one side of the culture medium, and fresh *B. cinerea* pieces with a diameter of 0.7 cm were inoculated on the other side of the culture medium. PDA plate without antagonistic yeast YE-3-2 was used as control. Three replicates were set for each yeast strain YE-3-2 and cultured at 20°C for 3 days. The colony radius (cm) of *B. cinerea* was measured.

#### 4.2.2. Statistical analysis

The experiment was repeated twice, with three replicates for each experiment. Excel 2010 was used for data collection and chart making. SPSS 22.0 was used to process the data. According to the inhibition rate, probability value, and logarithm of concentration, the sensitive baseline regression equation and EC_50_ value of inhibition medium concentration were obtained. In this experiment, when R^2^ > 0.7, the EC_50_ value is considered valid.

The Kolmogorov Smirnov test (K-S method) of SPSS software was used to test the normal distribution of EC_50_ value. When p > 0.05, it was considered normal distribution, otherwise, it was considered non-normal distribution. According to the principle of normal distribution of EC_50_ value of sensitive strains, when the EC_50_ value of tested strains was normal distribution, the average EC_50_ value was the sensitivity of the strain group to the drug Baseline; if the distribution is not normal, the average EC_50_ can not be regarded as the sensitive baseline of the pathogen population.

## 5. Result analysis

### 5.1. Identification results of gray mold resistance in grape population

**Figure 1.**
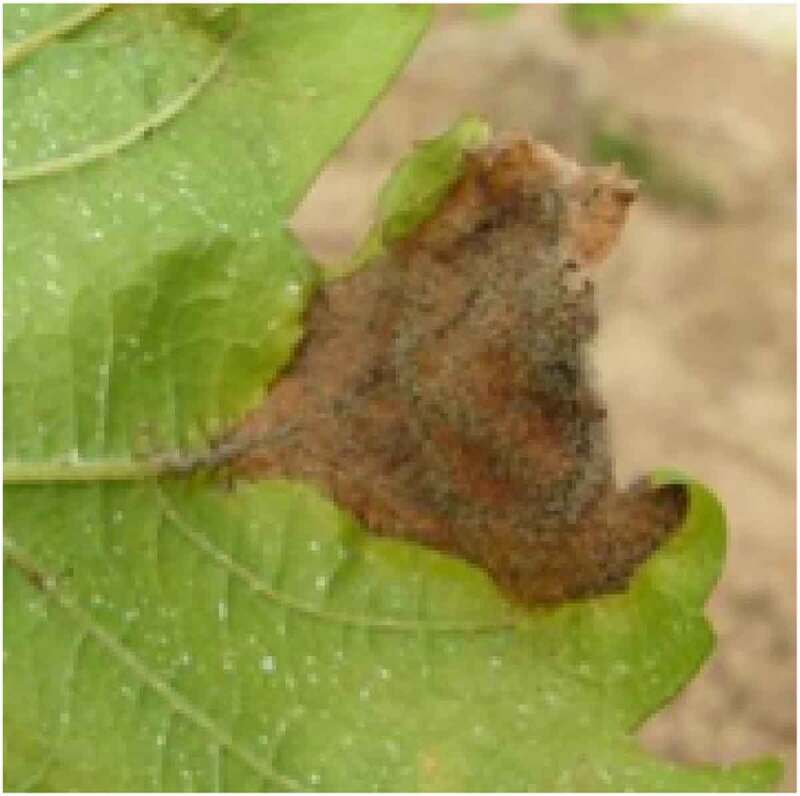
Disease state of grape leaves after inoculation of isolated leaves of single plant

**Figure 2.**
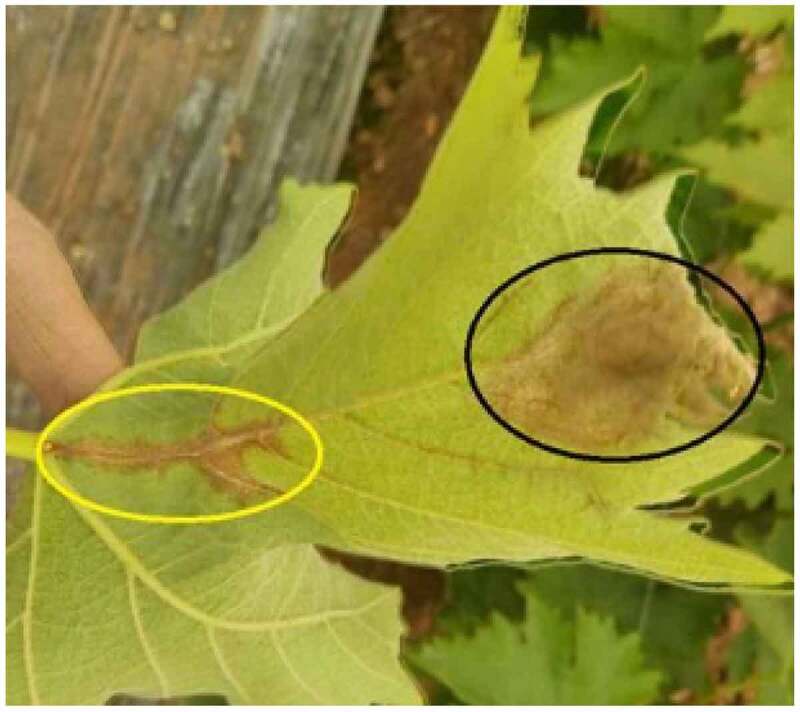
Incidence of single leaf infection of hybrid offspring in population after 5 days

The isolated leaves of the test population were inoculated with the cultured pathogen of grape gray mold. Figure 1 shows the disease state of grape leaves after inoculation of isolated leaves of single plant. Figure 2 shows the disease incidence of single plant leaves of hybrid progeny in the population after 5 days of infection. The results show that the disease incidence of single plant of two hybrid progeny populations is inconsistent, and the disease spot size is different in different degrees, which can reflect the polymorphism of disease resistance of the offspring population.

*B.cinerea* infects leaves, usually invading from thin leaves, forming ‘V’-shaped spots at leaf margins, which are irregular reddish brown. When the humidity is high, there are obvious gray mold layers. Look at the mold layers carefully, just like one grape spike. Gray mold infects leaves, and sometimes obvious disease spots appear on the veins, such as the yellow circle mark in the figure. However, when the humidity is low or after using chemicals correctly, the mold layer becomes not very obvious. Here, pay attention to distinguish the contrast between the mold layer in the yellow circle and the black circle. *B.cinerea* infects inflorescences, and the affected parts are rotten, with gray-black mold layers, and inflorescences without shedding flower caps are generally susceptible to *B. cinerea* when the humidity is high.

### 5.2. Culture results of grape gray mold pathogen

After 5 days of *B. cinerea* culture, hyphae around the culture medium grew vigorously, no spores were produced and hyphae were white, and no miscellaneous bacteria grew on the whole PDA culture medium, thus ensuring the purity of *Botrytis cinerea* and meeting the infection requirements.

### 5.3. *Detection of resistance of* B.cinerea *to main fungicides*

**Figure 3.**
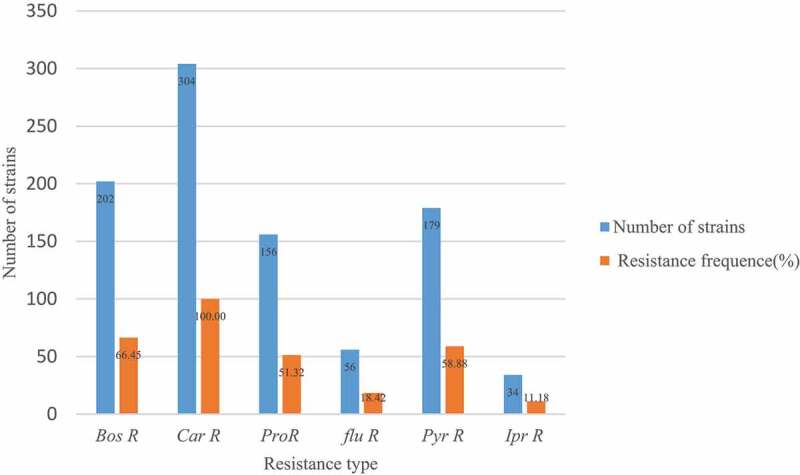
Resistance frequency of 304 *B.cinerea* strains to main fungicides

Among the 304 strains detected, 202 *boscalid* resistant strains (*Bos* R), accounting for 66.45%;146 strains were *carbendazim* resistant (*Car* R), accounting for 100%. 156 strains were *procymidone* resistant (*Pro* R), accounting for 51.32%; 56 strains were *fludioxonil* resistant (*flu* R), accounting for 18.42%; 179 *pyrimethanil* resistant strains (*Pyr* R), accounting for 58.88%;34 *iprodione* resistant strains (*Ipr* R), accounting for 11.18%; From the experimental data, the resistance frequency of 304 *B. cinerea* strains to *carbendazim, boscalid, pyrimethanil* was higher than 50% as shown in Figure 3.

### 5.4. *Resistance of* B. cinerea *to main fungicides in China*

**Figure 4.**
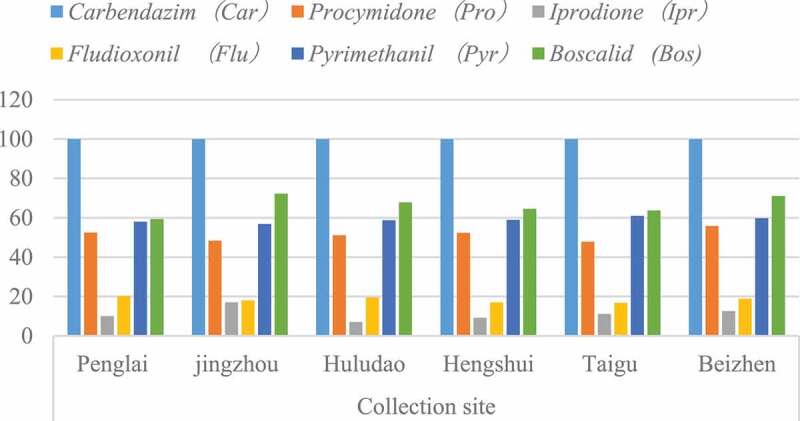
Resistance of *B. cinerea* to main fungicides in major grape-producing areas in China

From September 2015 to November 2019, 304 strains of *B. cinerea* were collected from Penglai, Shandong, Jingzhou, Hubei, Huludao, Liaoning, Hengshui, Hebei, Taigu, Shanxi, and Beizhen, Liaoning. The minimum inhibitory concentration method was used for *carbendazim, procymidone, iprodione* and *fludioxonil*, respectively. (see Figure 4, Table 1,2).

**Table 1.** Resistance of *Botrytis cinerea* to main fungicides in major grape-producing areas in China

		Resistance frequency/%					
Collection site	Total strains	*Carbendazim* (Car)	*Procymidone* (Pro)	*Iprodione* (Ipr)	*Fludioxonil* (Flu)	*Pyrimethanil* (Pyr)	*Boscalid* (Bos)
Penglai	36	100	52.51	10.03	20.19	57.98	59.32
jingzhou	29	100	48.39	17.03	17.98	56.86	72.21
Huludao	20	100	51.11	7.03	19.58	58.75	67.87
Yinchuan	101	100	52.32	9.24	17.01	58.99	64.51
Taigu	93	100	47.79	11.11	16.82	60.94	63.74
Beizhen	25	100	55.81	12.62	18.91	59.78	71.07

**Table 2.** Antibacterial activity of yeast against 304 strains of *Botrytis cinerea* in different climate planting areas

Grape climate planting area	Number of strains	Average bacteriostatic rate (%)
Cold zone	45	63.20
Cool and warm zone	101	60.30
Hot and humid area	129	65.53
Hot area	29	62.68
total	304	62.93

### 5.6. Resistance of B. cinerea to yeast (Trichosproom sp.) in China

The volatile compounds produced by yeast (*Trichosproom* sp.) strain YE-3-2 could inhibit 304 strains of *B. cinerea* in varying degrees, with the inhibition rate of 62.93%.

## 6. Occurrence regularity of grape gray mold

### 6.1. *Spore capture dynamics of* B. cinerea

*B. cinerea* overwinters on bark and winter buds with mycelia, or overwinter on branches, stiff fruits and soil with sclerotia. The conidia formed after germination in the following spring infect the buds and inflorescences. There are three peaks of gray mold in a year, the first one is before and after flowering, from mid-May to early June, which mainly harms flowers and young fruits, often causes inflorescences to rot, dry, and fall off, and further infects ears and rachis; The second attack occurred from the color change to the mature stage of the fruit, and the pathogen was most likely to invade from the wound. The sunken disease spots appeared on the fruit grain and ear axis, and soon the ear was soft rot and the stem turned black, forming a rat gray mold layer; For the third time, in the process of postharvest storage, if it is not properly managed, gray mold will occur, with obvious rat gray mold layer, which will lead to fruit ear rot.

**Figure 5.**
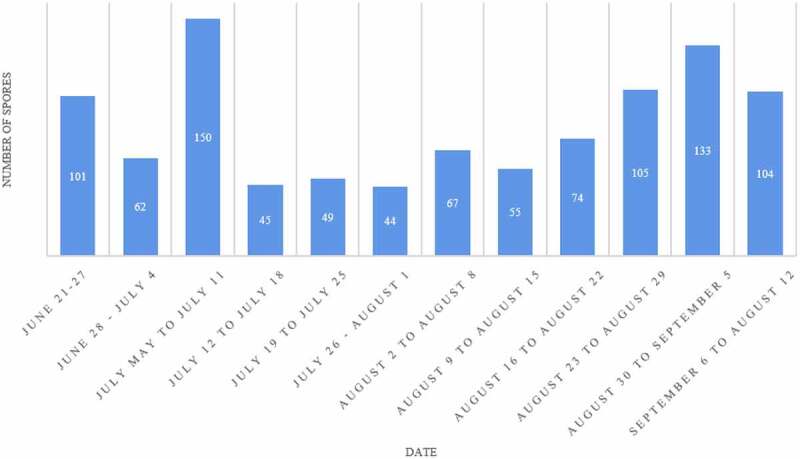
Dynamics of spore capture of grape downy mildew pathogen

In the growing season, the number of pathogen spores of *B. cinerea* on grape is more in the late stage than in the early stage, and the amount of spores scattered every day is different (Figure 5). The spore capture quantity of downy mildew was more from August to September, and the spore capture peak appeared in mid-August and early September. The first peak appeared on August 24th with 105 spores, the second peak appeared on August 30th with 133 spores, and the third peak appeared on September 6th with 104 spores.

The conditions for the occurrence of the disease are rainy, humid, and cool climate, and the suitable temperature for onset and epidemic is 15–23°C. In the foggy period, the spores of *B.cinerea* floated on the water vapor and drifted away with the wind, and landed on the leaves, buds, young shoots and young ears of grapes, and began to germinate and spread. The germs on the affected leaves splashed on other leaves along with the water drops on the leaves, causing the disease to spread.

### 6.2. The rate of grape gray mold in greenhouse changes with time

**Figure 6.**
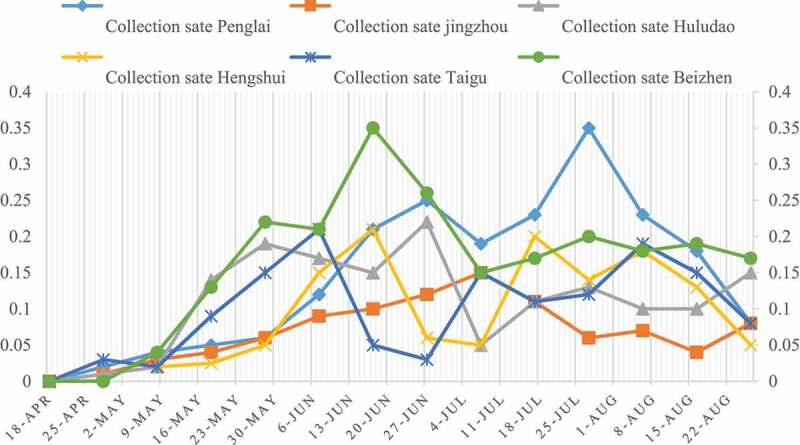
Changes of shed rate of grape gray mold disease with time

It can be seen from Figure 6 that *B. cinerea* showed a spreading trend from April 18 to may 28; From May 28 to July 7, the disease shed infected with gray mold remained basically unchanged; From July 7 to 27, the rate of disease shed infected with gray mold showed an upward trend again; From July 27 to August 16, the disease shed rate was basically in a stable state; After August 16th, the rate of sick shed began to decrease. The investigation found that the reason of ‘two liters’ is that grapes are easy to be infected with gray mold during the flowering stage and fruit color change stage, and they are often neglected in agricultural operations; The reason of ‘two stability’ is that grapes are not easy to be infected with gray mold at the expansion stage and maturity stage; The reason for the ‘one drop’ is that grapes are harvested at harvest time, and when the fruit farmers see the sick grapes, they remove them, which reduces the rate of sick shed.

The pathogen is a facultative parasite with a wide host range, which can infect not only grapes, but also up to 235 crops, such as tomatoes, cucumbers, eggplants, zucchini, strawberries, kidney beans, chrysanthemum organs, seedlings, fruits, and storage organs of apples and other plants [12]. The resistance frequency of mycoamine is higher than 90%. There are two peak periods of infection, that is, before flowering and after flowering, berries are colored to maturity; Third, the vineyard with low terrain, overgrown branches, poor ventilation and light transmission, extensive management, insufficient application of phosphorus and potassium, and serious pests is seriously ill; Before civilization, large and irregular reddish brown necrotic patches appeared on the leaves, which were mainly located at the edge of the leaves. Gray mold disease may not be observed on leaves at this time. It is very resistant to pesticides. Generally, one pesticide can only be sprayed once or twice. The more times, the worse the efficacy.

In autumn and winter, the diseased remains and pruned dead branches, fallen leaves, and diseased fruits in the field should be removed, burned, or buried deeply to reduce the source of germs. There are two peaks of gray mold outbreak in grape growth period, the first is from inflorescence separation to flowering period, which mainly harms inflorescence, causing it to rot, dry and fall off; The second time is from the coloring stage to the mature stage of grape, which is easy to cause fruit rot and mold layer. The disease is serious in low-lying orchards with high humidity, closed branches and leaves, low scaffolding, poor ventilation, and light transmission, poor drainage and easy to accumulate water, and it is easy to occur in vineyards with extensive management, insufficient fertilization, or more injuries (insect injury, mechanical injury, etc.).

### 6.3. Variation of flower spike rate of grape gray mold disease with time

**Figure 7.**
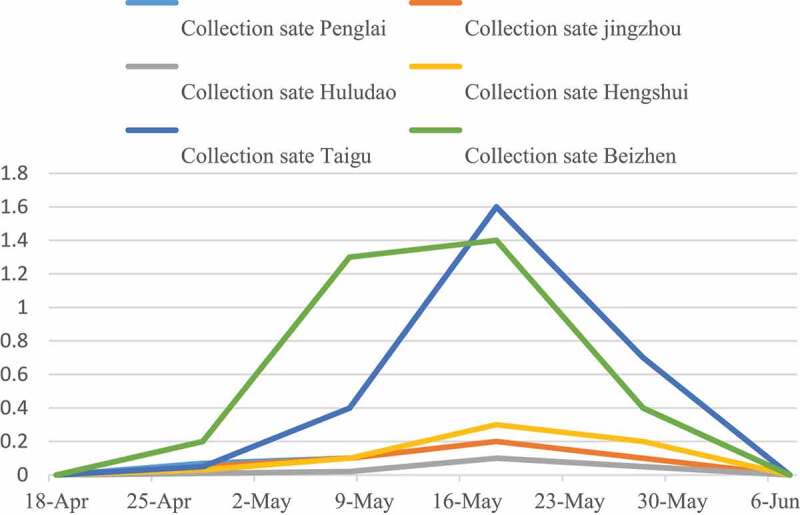
Variation of flower spike rate of grape gray mold disease with time

It can be seen from Figure 7 that the spike rate of gray mold disease showed an upward trend before May 18, and a downward trend after May 18. The reason for this phenomenon is that protected grapes are easily infected by *B. cinerea* at the flowering stage, and fruit farmers are busy with spring sowing in the field and neglect the management of greenhouse. With the passage of time, the number of flower spikes decreased, so did the infected flower spikes. The flower spike rate of Taigu County and Beizhen disease in six regions was significantly higher than that in the other three regions.

### 6.4. Changes of ear rate of grape gray mold disease with time

**Figure 8.**
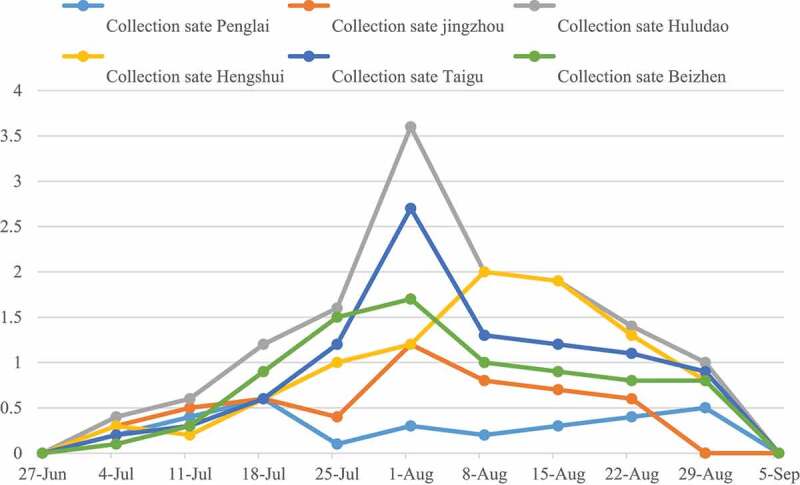
Changes of ear rate of grape gray mold disease with time

It can be seen from Figure 8 that the infection of grape *B. cinerea* to fruit ears showed an overall upward trend before August 6th, and the reason for this change trend was that the fruit was easily infected by *B. cinerea* during the color-changing period, and the grape grains were cracked due to inadequate management. On August 16th, the rate of diseased ears infected with *B. cinerea* showed a downward trend as a whole. The reason for this change was that the farmers began to clean the ears when the grapes went on the market, which reduced the rate of diseased ears. The fruit rate of Harbin gray mold was the highest and the lowest in six regions.

Its characteristics: first, the infection period is long, from germination to harvesting and storage, except that it is not easy to infect when the weather is dry; Second, there are two peak periods of infection, that is, before flowering to after flowering and after berry coloring to maturity; Grape over-dense planting, poor ventilation, unscientific fertilization, poor nitrogen, less phosphorus and potassium, poor drainage, and high humidity are all easy to cause gray mold. If it encounters low temperature and wet rainy weather, it will cause the peak of pathogen infection. After a short incubation, it will break out quickly after the temperature rises, which is difficult to control.

## 7. Pollution-free control measures of grape gray mold

### 7.1. Biological control

In ecological environment, microorganisms can inhibit the growth of other microorganisms through one or more mechanisms such as antagonism, parasitism, resistance induction, competition, and growth promotion. Different grape varieties have different resistance to gray mold. At present, the antagonistic bacteria against gray mold include *Trichoderma, Helicoverpa, Trichosporon, Bacillus, Leucocystin, allozyme, streptomycin*, etc. Using microorganisms to induce host plants to produce defensive response and form local or systemic acquired resistance, *Trichoderma* harzianum 300 times solution can be used for control. The bacterial agent Weijunjing made of *Bacillus* subtilis can control cotton wilt; Yield Shield, a microbial inoculum prepared from Bacillus pumilus, can be used to control soybean sheath blight.

The control and application of actinomycetes to gray mold are mainly *wuyiencin*, *phosphazenin*, *leucopeptidomycin* and so on [13]. Jia Shuangshuang. [14] studied The resistant strains aer the main ones ,when selecting fungicides in production ,try to avoid using three agents at the same time .The strain atrum13 can also colonize grape seedlings and promote their growth. However, at present, most of the conclusions on the control effect of antagonistic bacteria against *B. cinerea* come from the research results on the control effect of antagonistic bacteria against *B.cinerea* under laboratory conditions, and the application effect under natural conditions needs to be further studied.

### 7.2. Physical control

Han M M et al. [15] further studied the inhibition ability of ozone with concentration of 200–350 L/L on *B.cinerea* under different relative humidity conditions (35%, 75% and 95%), and found that when the environmental relative humidity was high, the mortality rate of *B.cinerea* conidia was high, and the infection rate of *B.cinerea* was reduced by ozone gas treatment (45–85%). Scientific shaping and pruning, thinning flowers and fruits, timely tying tendrils, thinning auxiliary shoots, coring, thinning leaves, and trimming ears, controlling the amount of branches left, increasing the fruiting position, adjusting the density of tendrils, improving ventilation and light transmission conditions, and reducing the infection chance of *B.cinerea*. In vineyards with severe diseases, it is generally better to use therapeutic agents and protective agents alternately. When using drugs for prevention and treatment, we should not only grasp the key period and treat the symptoms, but also ensure that the drugs are true and of high quality. The spraying should be uniform and thoughtful, with emphasis on the susceptible parts.

### 7.3. Comprehensive control measures

When building a garden, try to choose plots with high terrain and good ventilation. The row spacing should be relaxed appropriately, and the row direction should be parallel to the main wind direction. The scaffolding should not be too low to ensure ventilation and light transmission, which can effectively prevent or reduce the occurrence of gray mold.

Strengthen the management of fertilizer and water, apply more organic fertilizer, bio-bacterial fertilizer and chemical fertilizer, supplement medium and trace element fertilizer, avoid excessive input of nitrogen fertilizer, scientifically compound the ratio of nitrogen, phosphorus and potassium according to the nutrient demand law of grape in different growth stages, so as to improve the disease resistance of grape plants.

In autumn and winter, residues such as diseased leaves and stiff fruits are thoroughly removed and burned centrally. During the flowering period of grapes, when the dew is not dry in the morning, check and remove the diseased flower spikes, so as to reduce the bacteria source and disease center.

## 8. Discussion

At present, the reported pollution-free control methods of grape gray mold, especially biological control methods, are mostly limited to laboratory or field control experiments, and only a few have been commercialized. The shed rate of grape gray mold disease is 5–45%; The rate of diseased plants was 0.5–20.5%. The rate of diseased leaves was 0.02–0.35%. The rate of diseased spikes is 0–1.85%. The ear rate of diseased fruit is 0–5%. The onset time of grape gray mold is from April to August, and the two high incidence periods of gray mold are concentrated in May from flowering stage to young fruit stage; Because of the difference between indoor and outdoor environmental conditions, the mortality rate of the black-fronted brontispa is higher. In the experiment, only some habits of leaf beetle were recorded, and no more detailed data were obtained for its required temperature and humidity. *Carbendazim* and *pyrimethanil* have lost their control effects on *Botrytis cinerea* in almost all the investigated areas, which is closely related to the long-term, high-frequency and large-dose use of these two agents. It can be predicted that the control effects of *procymidone* and *iprodione* will become lower and lower with such use habits until they disappear completely. Zhang Yanjie studied on the morphology, pathogenicity, mating type, transposon genotype and microsatellite genetic diversity of *B.cinerea*, found that there is high genetic diversity in population of *B.cinerea*.[16]. In some areas, *dimethomorph* and *azoxystrobin* are still effective fungicides for controlling *B. cinerea*. The control effects of *dimethomorph* and *azoxystrobin* at three different concentrations are all over 90% 10 days after three treatments, so they can be used as main fungicides for controlling *B. cinerea* in the field. Zhang Zhengwei et al. [17] have even detected that the resistance frequency of *B.cinerea* to *fludioxonil* and boscalid in southeastern Germany is as high as 100%. This also shows that the control effect of *fludioxonil* will decrease rapidly with the increase of the number and amount of use in China, so prevention is the main priority in the field when the resistance of *fludioxonil* is not very common.

After the initial infection, many conidia will grow and infect again under the action of airflow. Other people’s studies show that gray mold resistance genes are controlled by polygenic loci, but to achieve fine location of gray mold resistance loci, it is necessary to carry out multi-point resistance identification for many years and analyze that exist stably in multiple environments [18]. Mancozeb is an excellent protective fungicide, which is a low-toxic pesticide. It has a wide range of sterilization and is not easy to produce resistance. Its mechanism of action is to inhibit the oxidation of pyruvate in bacteria; *Dimethomorph* is a new type of special low-toxic fungicide for systemic therapy. Its action mechanism is to destroy the formation of cell wall membrane of pathogen, cause the decomposition of sporangium wall, and make pathogen die; In order to slow down the generation of drug resistance in *B. cinerea* control, it is suggested to use different types of drugs alternately in production. In addition, in order to ensure excellent control effect, *B. cinerea* should be applied at the early stage of the disease, and should be used alternately at intervals of 7 days to 10 days.

In this study, the resistance of *B. cinerea* from Penglai Shandong Province, Jingzhou Hubei Province, Huludao Liaoning Province, Hengshui Hebei Province, Taigu Shanxi Province, and Beizhen Liaoning Province were tested. The results showed that the resistance frequency of *B. cinerea* in these eraes to *carbendazim* was up to 100%, and even most of them are higher than 50%. Chen Jin [19] found in the experiment that the volatile substances produced by Hansenula viticola can inhibit the growth of *B. cinerea*, cause the deposition of mycelium pigment and change the shape and size of mycelium; At the same time, it can inhibit the germination of spores and shorten the length of bud tube, but it can not completely kill the spores of *Vitis vinifera*. Comprehensive analysis of the results of gray mold identification shows that the isolated population has no obvious separation ratio, but presents continuity, which accords with the condition that the absolute values of skewness and kurtosis of normal distribution are also less than one; The identification results of this experiment showed that it was consistent with the genetic characteristics of quantitative traits and suitable for analysis.

## 9. Conclusion

This study preliminarily verified the bacteriostatic mechanism of yeast (*Trichosproom sp*.) against *B .cinerea*, but the molecular mechanism of bacteriostasis needs further study. At present, most strains or microbial agents are directly used for prevention and control in the market, and the shelf life and effect stability of the agents are difficult to guarantee, and the use cost is high. However, how to use volatile compounds produced by yeast (*Trichosproom sp*.) to control the storage diseases of fruits and vegetables is not perfect, and there is still a lack of practical application technology, which needs further verification, development, and popularization.

## Highlights

1. In this experiment, it was found that the volatile substances metabolized by this strain could effectively control botrytis cinerea
2. This study provides a safe and effective biological control way for controlling botrytis cinerea after grape harvest.
3. The phenotypic value of resistance could be used for QTL mapping analysis of gray mold resistance.
